# Empagliflozin does not change cardiac index nor systemic vascular resistance but rapidly improves left ventricular filling pressure in patients with type 2 diabetes: a randomized controlled study

**DOI:** 10.1186/s12933-020-01175-5

**Published:** 2021-01-07

**Authors:** Matthias Rau, Kirsten Thiele, Niels-Ulrik Korbinian Hartmann, Alexander Schuh, Ertunc Altiok, Julia Möllmann, András P. Keszei, Michael Böhm, Nikolaus Marx, Michael Lehrke

**Affiliations:** 1 Department of Internal Medicine I, University Hospital Aachen, RWTH Aachen University, Pauwelsstraße 30, 52074 Aachen, Germany; 2 Center for Translational & Clinical Research Aachen (CTC-A), RWTH Aachen University, Aachen, Germany; 3 Department of Internal Medicine III, University Hospital Saarland, Saarland University, Homburg/Saar, Germany

**Keywords:** SGLT2 inhibitors, Diabetes, Diastolic function, Hemodynamic parameters

## Abstract

**Background:**

In the EMPA-REG OUTCOME trial (Empagliflozin Cardiovascular Outcome Event Trial) treatment with the sodium-glucose cotransporter-2 (SGLT2) inhibitor empagliflozin significantly reduced heart failure hospitalization (HHF) in patients with type 2 diabetes mellitus (T2D) and established cardiovascular disease. The early separation of the HHF event curves within the first 3 months of the trial suggest that immediate hemodynamic effects may play a role. However, hitherto no data exist on early effects of SGLT2 inhibitors on hemodynamic parameters and cardiac function. Thus, this study examined early and delayed effects of empagliflozin treatment on hemodynamic parameters including systemic vascular resistance index, cardiac index, and stroke volume index, as well as echocardiographic measures of cardiac function.

**Methods:**

In this placebo-controlled, randomized, double blind, exploratory study patients with T2D were randomized to empagliflozin 10 mg or placebo for a period of 3 months. Hemodynamic and echocardiographic parameters were assessed after 1 day, 3 days and 3 months of treatment.

**Results:**

Baseline characteristics were not different in the empagliflozin (n = 22) and placebo (n = 20) group. Empagliflozin led to a significant increase in urinary glucose excretion (baseline: 7.3 ± 22.7 g/24 h; day 1: 48.4 ± 34.7 g/24 h; p < 0.001) as well as urinary volume (1740 ± 601 mL/24 h to 2112 ± 837 mL/24 h; p = 0.011) already after one day compared to placebo. Treatment with empagliflozin had no effect on the primary endpoint of systemic vascular resistance index, nor on cardiac index, stroke volume index or pulse rate at any time point. In addition, echocardiography showed no difference in left ventricular systolic function as assessed by left ventricular ejections fraction and strain analysis. However, empagliflozin significantly improved left ventricular filling pressure as assessed by a reduction of early mitral inflow velocity relative to early diastolic left ventricular relaxation (E/eʹ) which became significant at day 1 of treatment (baseline: 9.2 ± 2.6; day 1: 8.5 ± 2.2; p = 0.005) and remained apparent throughout the study. This was primarily attributable to reduced early mitral inflow velocity E (baseline: 0.8 ± 0.2 m/s; day 1: 0.73 ± 0.2 m/sec; p = 0.003).

**Conclusions:**

Empagliflozin treatment of patients with T2D has no significant effect on hemodynamic parameters after 1 or 3 days, nor after 3 months, but leads to rapid and sustained significant improvement of diastolic function.

*Trial registration* EudraCT Number: 2016-000172-19; date of registration: 2017-02-20 (clinicaltrialregister.eu)

## Background

Sodium-glucose cotransporter-2 (SGLT2) inhibitors are glucose-lowering drugs currently used to treat patients with type 2 diabetes mellitus (T2D). These agents act by inhibiting SGLT2 in the proximal tubule of the kidney with a subsequent increase in urinary glucose excretion thus lowering blood glucose levels. Several placebo-controlled cardiovascular outcome trials (CVOTs) with SGLT2 inhibitors (EMPA-REG OUTCOME with empagliflozin [1], the CANVAS program [2] and CREDENCE [3] with canaglifozin, DECLARE with dapagliflozin [4], VERTIS with Ertugliflozin [5]) demonstrated a reduction in CV events as well as a reduction in hospitalisation for heart failure (HHF) in patients with T2D and atherosclerotic CV disease (ASCVD), multiple CV risk factors, or diabetic nephropathy. Moreover, the favourable effects of SGLT2 inhibitors on HHF and CV death in these trials were present in patients with or without HF at baseline [6], suggesting that these agents could prevent the development of HF in patients with T2D. In addition, data from the DAPA-HF [7] and the EMPEROR reduced trial [8] suggest that SGLT2 inhibitors may reduce HF related endpoints and CV death even independent of the presence of diabetes.The underlying mechanisms of these beneficial effects of SGLT2 inhibitors on HF-related events remain unclear but changes in blood pressure, blood glucose, or body weight are unlikely to solely explain the observed results. The early separation of HHF event curves in the CVOTs suggested SGLT2 inhibition to provide immediate effects on volume status and/or modulation of hemodynamic parameters potentially mediated by early diuretic effects [9–13]. Therefore, we conducted a prospective, placebo-controlled, double blind, randomized, exploratory pilot study in patients with T2D to assess the effect of empagliflozin on urinary volume, left ventricular filling pressure and function in addition to hemodynamic parameters after 1 day, 3 days and 3 months of treatment.

## Methods

### Study population and study design

In this single center, prospective, placebo-controlled, double blind, randomized, 2-arm parallel, interventional and exploratory pilot study 44 patients with T2D were randomized into 2 groups. The randomisation list was computer generated using a permuted block randomisation with block size of 4. The sequence generation method and the block size was concealed from the investigators. An independent pharmacist labelled the study medications according to the randomisation list. Study participants received empagliflozin 10 mg or placebo for a period of 3 months in addition to their concomitant medication. Non-invasive hemodynamic measurement, transthoracic echocardiography, blood pressure, blood- and urine-chemistry were performed at baseline (day 0), day 1, day 3 and after 3 months. Participants were recruited from the Department of Internal Medicine I at University Hospital Aachen, RWTH Aachen University, Germany. Inclusion criteria were as follows: type 2 diabetes, HbA1c ≥ 6.5% and age ≥ 18 years. Exclusion criteria were type 1 diabetes, uncontrolled hypertension, age ≥ 85 years, pregnancy, renal impairment (eGFR < 30 mL/min/1.73 m^2^), liver disease (serum levels of AST, ALT or AP more than three times the upper limit of normal), uncontrolled thyroid disease, endocrinopathies like Graves’ disease, akromegaly, Cushings’ disease, secondary hypertension due to renal artery stenosis, pheochromocytoma or hyperaldosteronism, hypertensive retinopathy or encephalopathy, acute coronary syndrome, stroke or transient ischemic attack in last 6 weeks prior to randomization. The study protocol was approved by the local ethic committee and all subjects gave written informed consent. The trial was registered: EudraCT Number: 2016-000172-19.

### Laboratory measurement

Serum chemistry including haematology, lipid profile, glucose metabolism, eGFR (CKD-EPI formula), cystatin C, NT-proBNP, aldosterone were performed at every visit of the clinical trial. We collected 24 h urine at baseline, day 1, day 3 and after 3 months to measure renal excretion of glucose and sodium.

### Hemodynamics

We used ClearSight System® (Edwards Lifesciences, Irvine, USA) as a validated [14] non-invasive tool to explore effects of empagliflozin on hemodynamic parameters including cardiac index (CI), stroke volume index (SVI), heart rate (HR), and systemic vascular resistance index (SVRI) at baseline, day 1, day 3 and after 3 months. ClearSight System® uses finger arterial pressure measurement based on the volume clamp method in combination with Physiocal calibration. Dividing the systolic area of the time integral of the pressure curve above the diastolic pressure by the estimated arterial impedance gives a beat-to-beat stroke volume which is multiplied with the heart rate to reach cardiac output, as has been described previously [14].

### Transthoracic echocardiography

Transthoracic and Doppler echocardiography were performed by technicians blinded to clinical information and treatment assignment with commercially available ultrasound systems (GE Healthcare, Chicago, USA). Standardized echocardiographic measurements were obtained in accordance with the guidelines of the EACI (European Association of Cardiovascular Imaging) and ASE (American Society of Echocardiography). Left ventricular systolic function (EF) was measured in 4 chamber and 2 chamber views by Simpson’s Biplane Method. Additionally we performed myocardial deformation analysis of the left ventricle to assess peak global longitudinal strain (GLS) of the endocardial layer by speckle-tracking echocardiography in 4 chamber, 2 chamber and apical 3 chamber views. For diastolic function we determined early (E) and late (A) diastolic mitral inflow velocities, deceleration time (DT), septal early diastolic mitral annular tissue velocity (septal eʹ) and lateral early diastolic mitral annular tissue velocity (lateral eʹ) by mitral pulse wave Doppler and tissue Doppler. We calculated E/e' ratio and E/A ratio by dividing E peak by average eʹ calculated from septal eʹ and lateral eʹ respectively E peak by A. Additionally we performed myocardial deformation imaging as determined by 2D and 3D parameter global strain rate. Images were stored digitally for subsequent offline analysis. Interpretation of the echocardiograms was performed by two independent blinded investigators. Interobserver variability of the key echocardiographic endpoints E and eʹ was 0.8 for E and 0.77 for eʹ.

### Endpoints

The study was powered for primary study outcome of empagliflozin on systemic vascular resistance index (SVRI) in comparison to placebo after 1 day, 3 days and 3 months of treatment. Secondary endpoints included changes in the following parameters after 1 day, 3 days and 3 months: cardiac index (CI), stroke volume index (SVI), blood pressure, sodium excretion in 24 h urine collection, body weight, heart rate, serum levels of NT-proBNP, cystatin C, glucose, HbA1c and aldosterone.

Further secondary analysis included changes in left ventricular systolic function as determined by EF and GLS, and in left ventricular diastolic function as determined by standardized parameters.

### Statistical analysis

The sample size calculation was conducted based on a repeated measure analysis of variance of the primary endpoint including baseline and 3 repeated measures, 2 treatment levels, and a treatment-by-time interaction tested using an F-Test. A mean difference of zero at baseline and constant differences over time were assumed, and a standard deviation of 930 dynes s cm^−5^ m^−2^ was used based on sample standard deviation in previous work [15]. The correlation structure was assumed to follow compound symmetry with correlation of 0.3. A significance level of 5% and a power of 80% were chosen. Based on these assumptions, a total of 42 patients allows a detection of a minimal difference of 800 dynes s cm^−5^ m^−2^ in SVRI.

Descriptive statistics of baseline characteristics were calculated as relative (%) and absolute frequencies for categorical variables. Quantitative variables were described as means and standard deviations, in case of non-normally distributed data, as median with 1st and 3rd quartiles. Data distributions were visualized using box-plots.

Outcome variables were analysed using linear mixed models with fixed effects for treatment, visits (day 1, day 3 and 3 months) and baseline measurement of the variable. For the primary endpoint analysis, randomisation blocks were also included as fixed effect. The random part of the models consisted of intercepts grouped by individuals. Restricted maximum likelihood estimation was used. For NT-proBNP the log transformed variable was used in the analyses. Treatment effects were estimated at each visit along with Wald type 95% confidence intervals. For the primary endpoint the null hypothesis that all treatment-visit interactions are zero was tested against the alternative that at least one of them is not zero using an F test. Kenward–Roger approximation of the degrees of freedom was used. As additional analyses, correlation between changes from baseline to 3 months were calculated for selected variables using the Pearson correlation coefficient, and changes from baseline were compared between treatment groups separately at each visit. Results were not adjusted for multiple comparison.

## Results

### Baseline characteristics

From May 2017 to January 2019 a total of 44 patients underwent randomization. Data analysis was performed on 42 patients with 2 patients in the empagliflozin group being excluded because of protocol violations (concomitant intake of SGLT2 inhibitors at baseline and throughout study). No difference in baseline characteristics were observed between empagliflozin and placebo treated patients. Mean age of study participants was 62 ± 6.8 years, 81% were male, with a mean glycated hemoglobin of 7.7 ± 1.1%, a mean BMI of 31.3 ± 4.6 kg/m^2^, a mean eGFR of 83 ± 19 mL/min/1.73 m^2^, a history of CVD in 71%, and presence of chronic heart failure in 43% of all patients. Patients had a baseline blood pressure of 135/81 mmHg (SD 16.9/13.2) and a mean LDL cholesterol of 99 ± 36.9 mg/dL. Baseline medication was not different in both groups including anti-diabetic drugs, RAAS-inhibition, beta blockers and statins (Table 1).

**Table 1:** Baseline characteristics of the study population

	Placebo (N = 22)	Empagliflozin (N = 20)	p
Age—years	61.2 ± 7.9	62.8 ± 5.4	0.466
Male—no. (%)	18 (81.8)	16 (80)	0.881
BMI—kg/m^2^	31.2 ± 4.0	31.4 ± 5.3	0.905
Systolic blood pressure—mmHg	136 ± 18	135 ± 16	0.934
Diastolic blood pressure—mmHg	81 ± 14	82 ± 13	0.964
Heart rate—bpm	69 ± 15	71 ± 12	0.587
Type 2 diabetes			
Glycated hemoglobin—%	7.9 ± 1.3	7.5 ± 0.9	0.228
Diabetes duration—years	9 (6–18)	10 (4–14)	0.523
Insulin treated—no. (%)	8 (36)	11 (55)	0.475
Metformin—no. (%)	18 (82)	13 (65)	0.410
DPP-4 inhibitors—no. (%)	6 (27)	8 (40)	0.552
Others—no. (%)	1 (5)	3 (15)	0.308
History of CVD—no. (%)			
Coronary heart disease	15 (68.2)	15 (75)	0.625
Myocardial infarction	10 (45.5)	5 (25)	0.205
CABG	4 (18.2)	4 (20)	0.881
PCI	12 (54.5)	10 (50)	0.768
Peripheral artery disease	2 (9.1)	4 (20)	0.313
Chronic heart failure—no. (%)	11 (50)	7 (35)	0.327
Medication—no. (%)			
Antiplatelets	16 (73)	11 (55)	0.975
Oral anticoagulants	5 (23)	6 (30)	0.914
Diuretics	10 (45)	10 (50)	0.549
Statins	15 (68)	15 (75)	0.455
Calcium channel blockers	5 (23)	4 (20)	0.637
Beta blockers	16 (73)	16 (80)	0.345
RAAS inhibitors	20 (91)	15 (75)	0.115
e GFR—mL/min/1.73 m^2^	88 ± 16	77 ± 21	0.076
Total cholesterol—mg/dL	155 ± 39	169 ± 41	0.257
LDL-C—mg/dL	95 ± 38	103 ± 36	0.522
HDL-C—mg/dL	44 ± 9	43 ± 9	0.530
Triglycerides—mg/dL	156 ± 71	245 ± 150	*0.023*

Values are mean ± SD for normally distributed data and median and interquartile range for non-normally distributed data, or no. (%); p-values for continuous variables were calculated using t test, the p-value for diabetes duration was assessed by Kruskal–Wallis test; p-values for categorical variables were calculated using chi-squared test; p-values ≤ 0.05 were categorized as statistically significant

*BMI* body mass index, *CABG* coronary artery bypass graft, *CVD* cardiovascular disease, *eGFR* estimated glomerular filtration rate, *HDL-C* high density lipoprotein cholesterol, *LDL-C* low density lipoprotein cholesterol, *PCI* percutaneous coronary intervention, *RAAS* renin–angiotensin–aldosterone system

### Effect of empaglifozin on hemodynamic parameters

**Fig. 1:**
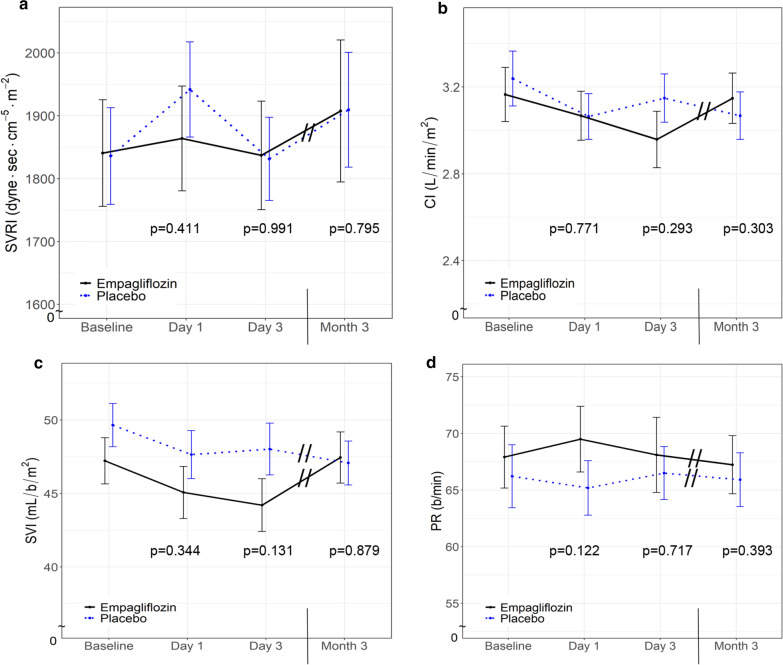
Hemodynamic parameters. Systemic vascular resistance index (SVRI) (**a**), cardiac index (CI) (**b**), stroke volume index (SVI) (**c**), and heart rate (HR) (**d**) in patients with type 2 diabetes treated with empagliflozin (n = 20; black line) or placebo (n = 22; blue dotted line). Data are shown as mean ± standard error at baseline, after 1 day, 3 days, and 3 months. p-values are calculated from Wald tests for the intervention effect at each visit

Systemic vascular resistance index (SVRI) and cardiac index (CI) were assessed as primary outcomes by non-invasive pulse wave contour analysis (ClearSight System®) with no significant difference between empagliflozin and placebo treated patients at any time point (Fig. 1a, b, Table 2). No treatment dependent difference in left ventricular stroke volume index (SVI) or pulse rate (PR) was observed (Fig. 1c, d, Table 2). Over time, blood pressure was reduced in empagliflozin-treated participants but the effect did not reach statistical significance (Table 2).

**Table 2:** Comparison of laboratory values, 24 h urine, body weight, hemodynamics, blood pressure and echocardiography during the study

	Baseline			Day 1			Day 3			Month 3		
	Placebo	Empagliflozin	p	Placebo	Empagliflozin	p	Placebo	Empagliflozin	p	Placebo	Empagliflozin	p
Laboratory												
Glucose—mg/dL	175 ± 52	181 ± 75	0.742	171 ± 48	152 ± 38	*0.049*	166 ± 52	146 ± 35	*0.037*	156 ± 52	151 ± 42	0.754
HbA1c—%	7.9 ± 1.3	7.5 ± 0.9	0.228	7.9 ± 1.3	7.4 ± 0.9	0.722	7.9 ± 1.2	7.4 ± 0.9	0.690	7.8 ± 1.5	7.1 ± 0.7	0.595
Total cholesterol—mg/dL	155 ± 39	169 ± 41	0.257	155 ± 37	174 ± 43	0.178	152 ± 40	168 ± 39	0.824	152 ± 42	185 ± 48	*0.001*
LDL-C—mg/dL	95 ± 38	103 ± 36	0.522	94 ± 36	102 ± 36	0.915	93 ± 39	102 ± 40	0.477	89 ± 39	112 ± 47	< *0.001*
HDL-C—mg/dL	44 ± 9	43 ± 9	0.530	44 ± 9	42 ± 10	0.947	44 ± 9	43 ± 9	0.900	46 ± 11	46 ± 10	0.937
eGFR—mL/min/1.73 m^2^	88 ± 16	77 ± 21	0.076	85 ± 16	70 ± 19	*0.014*	85 ± 17	70 ± 21	*0.039*	85 ± 16	68 ± 20	0.108
Cystatin C—mg/L	1.0 ± 0.2	1.2 ± 0.4	0.148	1.0 ± 0.2	1.3 ± 0.4	< *0.001*	1.0 ± 0.2	1.3 ± 0.4	*0.001*	1.0 ± 0.2	1.3 ± 0.4	< *0.001*
NT-proBNP—pg/mL	166 (73–238)	239 (91–463)	0.481	168 (67–252)	192 (63–385)	0.224	147 (58–226)	173 (57–402)	0.408	158 (42–262)	133 (32–500)	0.723
Aldosterone—pg/mL	83 ± 33	104 ± 65	0.213	88 ± 32	111 ± 67	0.825	96 ± 50	108 ± 59	0.635	108 ± 71	137 ± 104	0.522
24 h urine												
Urinary volume—mL/24 h	1788 ± 756	1740 ± 601	0.829	1626 ± 681	2112 ± 837	*0.011*	2007 ± 913	2111 ± 758	0.429	1664 ± 594	2319 ± 873	*0.001*
Glucose excretion—g/24 h	10.9 ± 22.7	7.3 ± 22.7	0.617	6.9 ± 14.1	48.4 ± 34.7	< *0.001*	7.5 ± 14.5	65.7 ± 43.3	< *0.001*	10.2 ± 18.7	67.6 ± 50.9	< *0.001*
Sodium excretion—mmol/24 h	196 ± 84	164 ± 88	0.255	181 ± 76	185 ± 111	0.223	203 ± 107	181 ± 126	0.970	175 ± 55	201 ± 145	0.054
Electrolyte-free water clearance—mL/24 h	− 15 ± 721	166 ± 830	0.467	− 124 ± 654	417 ± 802	*0.011*	90 ± 874	461 ± 551	0.070	− 91 ± 597	380 ± 765	*0.013*
Body weight—kg	94.0 ± 14.0	95.8 ± 17.3	0.724	94.1 ± 14.1	94.9 ± 17.0	*0.044*	94.0 ± 14.1	94.5 ± 17.2	*0.007*	93.7 ± 14.3	96.0 ± 19.6	0.059
Hemodynamics												
CI—L/min/m^2^	3.2 ± 0.6	3.2 ± 0.6	0.682	3.1 ± 0.5	3.1 ± 0.5	0.771	3.1 ± 0.5	3.0 ± 0.6	0.293	3.1 ± 0.5	3.1 ± 0.5	0.303
SVI—mL/b/m^2^	50 ± 7	47 ± 7	0.266	48 ± 8	45 ± 8	0.344	48 ± 8	44 ± 8	0.131	47 ± 7	47 ± 7	0.879
SVRI—dyne*s*cm^−5^*m^−2^	1836 ± 361	1841 ± 379	0.967	1942 ± 355	1864 ± 373	0.411	1831 ± 310	1837 ± 376	0.991	1909 ± 428	1908 ± 451	0.795
HR—bpm	66 ± 13	68 ± 12	0.665	65 ± 11	69 ± 13	0.122	66 ± 11	68 ± 14	0.717	66 ± 11	67 ± 10	0.393
Blood pressure												
Systolic—mmHg	136 ± 18	135 ± 16	0.934	134 ± 16	128 ± 16	0.279	133 ± 19	125 ± 16	0.115	132 ± 20	128 ± 15	0.318
Diastolic—mmHg	81 ± 14	82 ± 13	0.964	80 ± 13	80 ± 11	0.876	80 ± 12	80 ± 9	0.925	82 ± 11	79 ± 11	0.197
Echocardiography												
LV-EF—%	48 ± 6.8	51 ± 5.0	0.183	48 ± 6.2	51 ± 4.6	0.852	48 ± 6.1	51 ± 4.7	0.333	48 ± 6.4	51 ± 4.4	0.375
LVEDD—mm	50 ± 5	49 ± 5	0.365	50 ± 6	49 ± 5	0.864	49 ± 5	48 ± 5	0.449	50 ± 6	48 ± 6	0.994
LVESD—mm	36 ± 8	34 ± 6	0.386	36 ± 9	35 ± 5	0.595	35 ± 8	34 ± 5	0.187	37 ± 8	33 ± 6	0.284
IVSd—mm	10 ± 2	10 ± 1	0.940	10 ± 2	11 ± 2	0.764	10 ± 2	11 ± 2	0.630	10 ± 2	10 ± 1	0.497
LV mass index—g/m^2^	91 ± 21	86 ± 19	0.474	94 ± 24	87 ± 19	0.448	90 ± 21	84 ± 19	0.701	89 ± 23	84 ± 17	0.807
LA area—cm^2^	20 ± 4.2	20 ± 3.7	0.484	20 ± 5.3	18 ± 4.3	0.082	20 ± 5.7	19 ± 4.8	0.691	20 ± 4.1	18 ± 4.0	0.503
LA volume index—mL/m^2^	31 ± 11	28 ± 9	0.414	30 ± 13	26 ± 10	0.300	30 ± 13	28 ± 10	0.840	31 ± 12	26 ± 9	0.239
E—m/s	0.78 ± 0.14	0.80 ± 0.20	0.686	0.80 ± 0.12	0.73 ± 0.20	*0.003*	0.78 ± 0.11	0.72 ± 0.18	*0.005*	0.78 ± 0.13	0.72 ± 0.21	*0.001*
A—m/s	0.73 ± 0.17	0.82 ± 0.17	0.095	0.74 ± 0.20	0.81 ± 0.15	0.786	0.75 ± 0.19	0.80 ± 0.15	0.550	0.73 ± 0.20	0.83 ± 0.15	0.927
E/A	1.18 ± 0.53	0.97 ± 0.22	0.121	1.20 ± 0.55	0.88 ± 0.20	*0.042*	1.16 ± 0.58	0.89 ± 0.23	0.181	1.21 ± 0.52	0.85 ± 0.23	*0.038*
eʹ septal—cm/s	7.2 ± 1.7	7.5 ± 1.9	0.603	6.6 ± 2.0	7.8 ± 2.1	0.077	7.2 ± 1.6	7.5 ± 2.0	0.785	7.2 ± 1.6	7.8 ± 2.1	0.470
eʹ lateral—cm/s	9.8 ± 2.0	10.4 ± 1.8	0.319	9.6 ± 2.4	10.1 ± 2.4	0.970	9.5 ± 1.9	9.8 ± 2.3	0.840	9.3 ± 2.0	10.4 ± 2.9	0.262
eʹ mean	8.5 ± 1.5	8.9 ± 1.6	0.361	8.1 ± 1.8	8.9 ± 2.1	0.352	8.4 ± 1.3	8.7 ± 2.0	0.968	8.3 ± 1.5	9.1 ± 2.3	0.280
E/eʹ mean	9.3 ± 2.2	9.2 ± 2.6	0.898	10.1 ± 1.4	8.5 ± 2.2	*0.005*	9.5 ± 1.5	8.5 ± 2.4	0.079	9.7 ± 1.9	8.3 ± 2.9	*0.004*
DT—msec	198 ± 54	206 ± 45	0.610	189 ± 45	212 ± 35	0.234	202 ± 54	215 ± 43	0.624	196 ± 59	218 ± 59	0.261
RVSP—mmHg + CVP	28 ± 6	29 ± 4	0.515	26 ± 5	26 ± 6	0.827	25 ± 4	29 ± 10	0.261	27 ± 8	26 ± 11	0.632
GLS (endocardial layer)	− 17 ± 5.3	− 19 ± 4.1	0.107	− 17 ± 4.8	− 19 ± 3.4	0.877	− 17 ± 4.4	− 19 ± 3.7	0.735	− 17 ± 4.6	− 19 ± 2.7	0.608

Values are mean ± SD for normally distributed data and median and interquartile range for non-normally distributed data; p-values at baseline were calculated using t test, the p-value for NT-proBNP was assessed by Kruskal–Wallis test; p-values for the intervention effect at day 1, day 3 and month 3 were calculated using the Wald method, eʹ mean is the mean of eʹ septal and eʹ lateral; p-values ≤ 0.05 were categorized as statistically significant

*bpm* beats per min, *CI* cardiac index, *CVP* central venous pressure, *DT* deceleration time, *eGFR* estimated glomerular filtration rate, *GLS* global longitudinal strain, *HDL-C* high density lipoprotein cholesterol, *HR* heart rate, *IVSd* interventricular septum in diastole, *LA* left atrial, *LDL-C* low density lipoprotein cholesterol, *LV* left ventricular, *LVEDD* left ventricular enddiastolic diameter, *LV-EF* left ventricular ejection fraction, *LVESD* left ventricular endsystolic diameter, *RVSP* right ventricular systolic pressure, *SVI* stroke volume index, *SVRI* systemic vascular resistance index

### Effect of empagliflozin on metabolic parameters and renal function

**Fig. 2:**
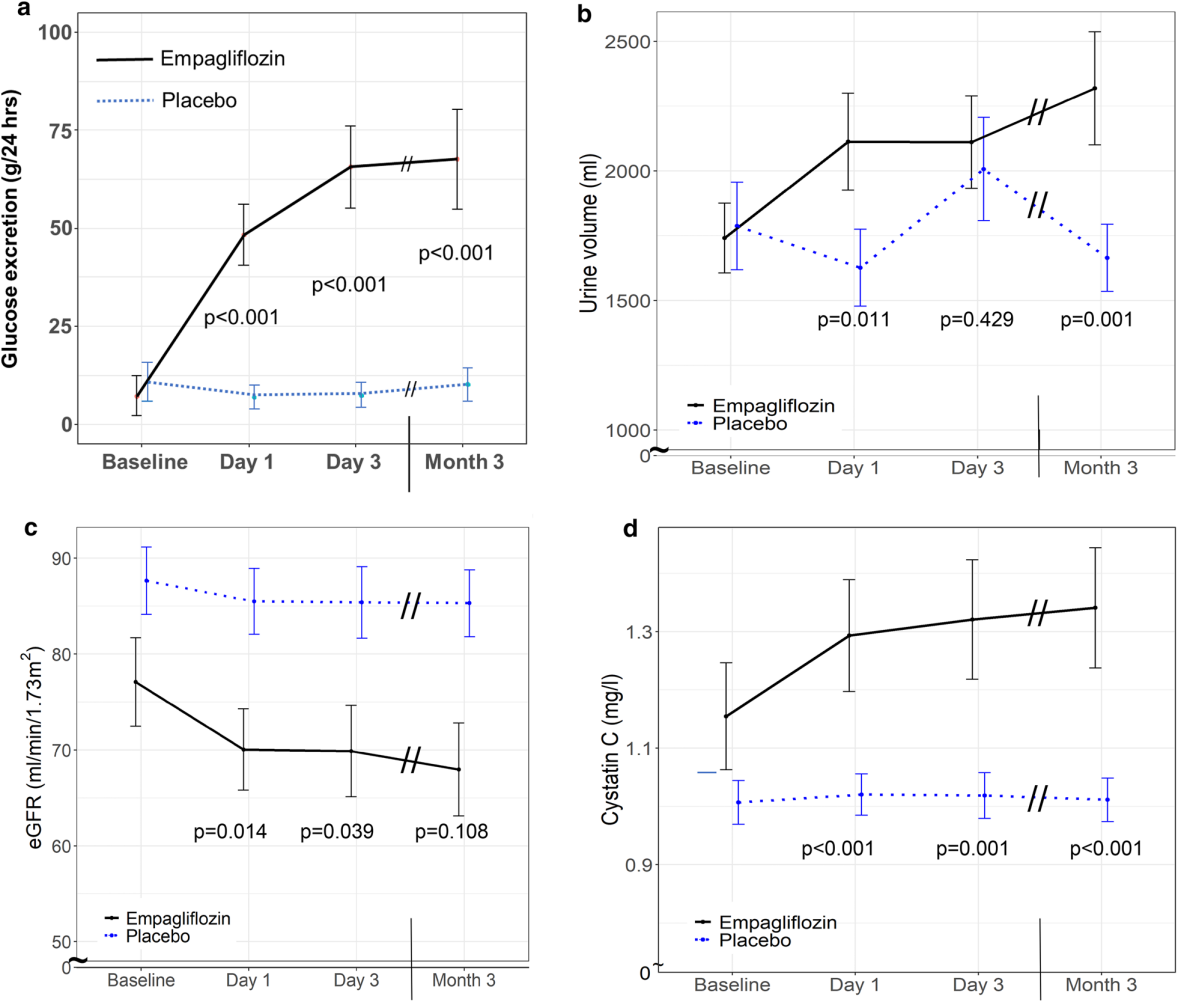
Metabolic parameters and renal function. Urinary glucose excretion (**a**), 24 h urinary volume (**b**), eGFR (**c**), and plasma cystatin C levels (**d**) in patients with type 2 diabetes treated with empagliflozin (n = 20; black line) or placebo (n = 22; blue dotted line). Data are shown as mean ± standard error at baseline, after 1 day, 3 days, and 3 months. p-values are calculated from Wald tests for the intervention effect at each visit

As expected, empagliflozin treatment significantly increased urinary glucose excretion already after one day from 7.3 ± 22.7 g/24 h to 48.4 ± 34.7 g/24 h (p < 0.001) (Fig. 2a and Table 2) which led to an early decrease of fasting blood glucose levels from 181 ± 75 mg/dL to 152 ± 38 mg/dL (p = 0.049) (Table 2). Urinary volume significantly expanded in parallel with glucosuria after day 1 from 1740 ± 601 mL/24 h to 2112 ± 837 mL/24 h (p = 0.011) and remained significantly increased after 3 months of treatment (2319 ± 873 mL/24 h; p = 0.001) compared to placebo (Fig. 2b and Table 2). Body weight decreased at day 1 and day 3 of empagliflozin treatment, which was however not sustained after 3 months (Table 2). We did not find a significant correlation between body weight reduction and urinary volume excretion.

Consistent with the initiation of renal tubule-glomerular feedback, empagliflozin significantly decreased eGFR from 77 ± 21 mL/min/1.73 m^2^ at baseline to 70 ± 19 mL/min/1.73 m^2^ (p = 0.014) after 1 day of treatment (Fig. 2c and Table 2) and increased serum cystatin C compared to placebo (Fig. 2d and Table 2). 24 h urinary sodium excretion increased in empagliflozin-treated patients without reaching statistical significance (Table 2). In addition, empagliflozin increased electrolyte-free water clearance from 166 ± 830 mL/24 h at baseline to 417 ± 802 mL/24 h after 1 day (p = 0.011), an effect that was sustained over the 3 month study period (Table 2).

### Effect of empagliflozin on echocardiographic parameters

**Fig. 3:**
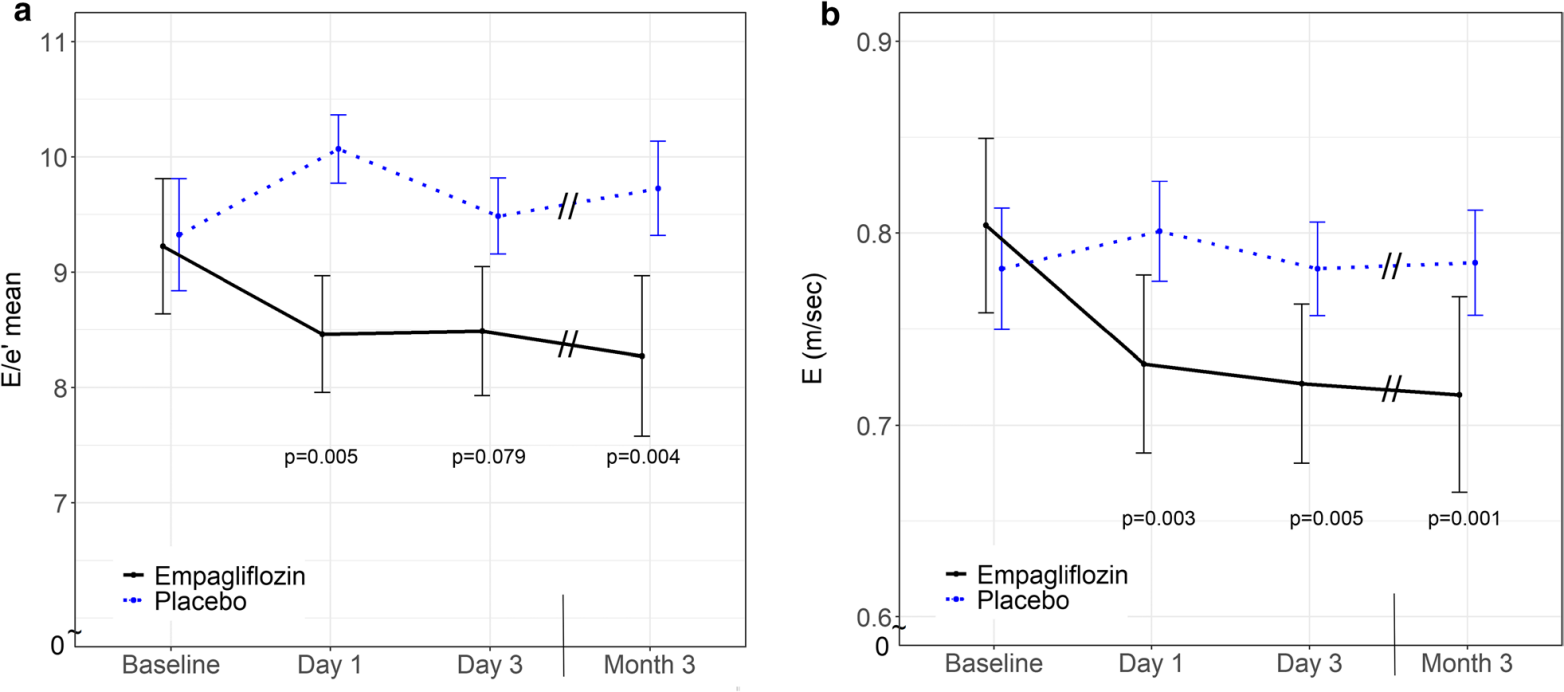
Left ventricular diastolic function. Early mitral inflow velocity relative to early diastolic left ventricular relaxation (E/eʹ) in patients with type 2 diabetes treated with empagliflozin (n = 20; black line) or placebo (n = 22; blue dotted line). Data are shown as mean ± standard error at baseline, after 1 day, 3 days, and 3 months. p-values are calculated from Wald tests for the intervention effect at each visit

Empaglifozin did not affect left ventricular systolic function as indicated by unchanged left ventricular EF and GLS values (Table 2). However, empagliflozin significantly improved left ventricular diastolic function as assessed by early mitral inflow velocity relative to early diastolic left ventricular relaxation (E/eʹ) which became significant at day 1 of treatment (baseline: 9.2 ± 2.6; day 1: 8.5 ± 2.2; p = 0.005) and remained apparent throughout the study (Fig. 3a, Table 2). Moreover, empagliflozin treatment significantly reduced early mitral inflow velocity (E) (baseline: 0.80 ± 0.20 m/s; day 1: 0.73 ± 0.20 m/s; p = 0.003) (Fig. 3b), but no differences were observed for early diastolic left ventricular relaxation (eʹ) (Table 2). Further analyses did not detect significant treatment dependent effects on left ventricular mass index, atrial volume index (Table 2), NT-proBNP or aldosterone, levels between groups during the 3 months treatment period (Table 2).

Changes in E/eʹ did not correlate with changes in urinary volume, urinary glucose or sodium excretion, left ventricular mass index or electrolyte-free water clearance (Additional file 1: Figure S1).

### Safety

A similar proportion of patients experienced adverse events in the empagliflozin and placebo arm of the study while more patients receiving empagliflozin experienced serious adverse events (Table 3). Genital infections and events consistent with volume depletion were reported more often from patients with empagliflozin (Table 3).

**Table 3:** Safety and adverse events

	Placebo (N = 22)	Empagliflozin (N = 20)
Adverse events	10	5
Serious adverse events	4	6
Hypoglycemic events	2	4
Death	0	0
Adverse event leading to discontinuation of a study drug	0	1
Acute renale failure	0	0
Event consistent with volume depletion	0	3
Thromboembolic event	0	1
Diabetic ketoacidosis	0	0
Urinary tract infection	0	1
Genital infection	0	4
Bone fracture	0	0

## Discussion

In this randomized, placebo-controlled, double-blind study in patients with T2D and prevalent ASCVD or high CV risk, resembling the populations studied in CVOTs with SGLT2 inhibitors, empagliflozin had no significant effect on hemodynamic parameters including systemic vascular resistance index, cardiac index, stroke volume indexor pulse rate after 1 or 3 days of treatment nor after 3 months. These data suggest that the early reduction in HF hospitalization seen in EMPA-REG OUTCOME [1], the CANVAS program [2], CREDENCE [16],DECLARE [4] and VERTIS [5] is unlikely to be caused by changes in these parameters. However, we found a rapid improvement in left ventricular filling pressure as shown by a reduction of early mitral inflow velocity relative to early diastolic left ventricular relaxation (E/eʹ) as a main measure of diastolic function, an effect already significant after one day of treatment and sustained until the end of the study. This was attributable to reduced early diastolic transmitral inflow (E), most likely a consequence of persistently increased diuresis induced by empagliflozin being apparent throughout the whole study period, whereas no difference was observed for early diastolic left ventricular relaxation.

The lack of a hemodynamic response to SGLT2 inhibition seen here differs from the response to classic diuretic drugs like loop diuretics. Acutely, loop diuretics increase urine excretion by reducing intravasal volume with apparent hemoconcentration and the diuretic-induced preload reduction impairs cardiac output with a compensatory increase in pulse rate and systemic vascular resistance [17]. In contrast, SGLT2 inhibition in our study rapidly expanded urinary volume excretion already after one day—along with an increase in electrolyte-free water clearance—which did not effect cardiac index, systemic vascular resistance nor pulse rate. Furthermore, treatment with empagliflozin did not decrease serum sodium levels as a common side effect of loop diuretics—with hyponatraemia being a powerful predictor of mortality in patients with heart failure [18]. So, it has been suggested that SGLT2 inhibition more efficiently reduces interstitial relative to intravasal volume in comparison to loop diuretics [19], which maybe supported by increased electrolyte free water clearance upon empagliflozin treatment in our study. Early effects of empagliflozin on body fluid content was further suggested by significant reduction of body weight at day 1 and 3 of treatment, which was however not sustained at the 3 month time point despite ongoing diuretic efficacy. Consistently Schork et al. reported rapid loss of extracellular water by SGLT2 inhibition using bioimpedance spectroscopy [20], which was not anymore apparent after 3 months of treatment. This suggests adaptive mechanisms of fluid regulation to compensate for the ongoing loss of urinary volume at later time points. Furthermore this might indicate additional mechanisms to be of relevance for the sustained reduction of heart failure events in respective CVOTs [1–5]. Importantly, SGLT2 inhibition has recently been found to reduce heart failure events to a similar extend in patients with and without diabetes demonstrating broad therapeutic efficacy of the drug class in HFrEF [7, 8].

The main—albeit exploratory—finding of our study, the early and sustained improvement of left ventricular filling pressure as indicated by E/eʹ in empagliflozin treated patients might provide important information to better understand the early beneficial effects on HF hospitalization seen in SGLT2 inhibitor outcome trials. Patient with T2D are at risk for diastolic dysfunction, resulting from increased left ventricular fibrosis, stiffness, and wall thickness as predisposing factors for heart failure with preserved ejection fraction. Consequential increase of left ventricular filling pressure causes augmentation of E/eʹ (early transmitral inflow velocity / early diastolic mitral annular tissue velocity) as an established echocardiographic parameter of diastolic dysfunction.

Given that impaired diastolic function is a crucial pathophysiological feature of HF, mainly subclinical HFpEF,—often present long before HF becomes clinical apparent—our data bolster the hypothesis that SGLT2 inhibitors could prevent the development of HF by improving left ventricular filling pressure in patients with T2D. An observatory study of 37 patients with T2D demonstrated a reduction in E/eʹ, which was also attributable to reduced early transmitral inflow velocity (E) and combined with a decrease in systolic blood pressure and LVMI after 3 months of treatment with canagliflozin [21]. In contrast, Soga et al. observed a decrease in E/eʹ unrelated to changes of blood pressure in 58 T2D patients with HF treated for 6 months with dapagliflozin under non-randomised conditions. In this study the improvement in diastolic function was independent of E, but attributable to reduced early diastolic left ventricular relaxation and paralleled by a reduction in LVMI [22]. Furthermore Higashikawa et al. reported Tofogliflozin to improve E/eʹ in 42 elderly patients with diabetes [23].

Our randomized, placebo-controlled study extends the understanding of SGLT2 inhibitors’ effect on diastolic function by demonstrating time dependent effects of empagliflozin on left ventricular filling pressure being apparent already after 1 day of treatment. This might be attributable to empagliflozin dependent osmotic diuresis with electrolyte free water excretion leading to cardiac preload reduction as suggested by reduced early mitral inflow velocity E. Still, modulation of E/eʹ did not correlate with changes in urinary volume, urinary glucose or sodium excretion, left ventricular mass index, electrolyte-free water clearance. Additional studies using larger populations will be required to clarify the relevance of volume unloading by SGLT2 inhibition for diastolic function. While observational studies suggest SGLT2 inhibition to improve outcome in patients with HFpEF, this is currently evaluated in large clinical trials (ClinicalTrials.gov Identifier: NCT03619213) [24, 25].

This study has certain limitations. First, hemodynamic parameters were assessed by non-invasive pulse contour analysis (ClearSight System®). However, this technique has extensively been validated against invasive hemodynamic measurements and is an established method in clinical practice [14, 26, 27]. Second, we did not measure other hemodynamic parameters such as pulse pressure, central arterial blood pressure, markers of arterial stiffness that have been shown to be affected by empagliflozin treatment for 6 weeks [28]. Third, the immediate improvement in diastolic function, shown by an early reduction of E/eʹ upon empagliflozin treatment, is an exploratory finding in a limited number of patients, and warrants confirmation in a larger study with changes in diastolic function defined as primary outcomes. Still, the present study was randomized, blinded and placebo-controlled, and changes in cardiac function assessed by echocardiography were predefined exploratory endpoints. Finally, improved diastolic function by empagliflozin treatment was not associated with reduced left ventricular mass index or reduced left atrial volume index nor RVSP after 3 months in our study, while others have found SGLT2 inhibition to reduce left ventricular mass [29]. Additional studies using larger populations will be required to investigate effect of SGLT2 inhibition on structural changes of the left ventricle.

## Conclusion

Taken together, our data suggest that empagliflozin treatment of patients with T2D and ASCVD/high CV risk leads to an immediate volume unloading and a rapid and sustained improvement of left ventricular filling pressure. These mechanisms could contribute to the early beneficial effects of SGLT2 inhibitors on HF hospitalisation seen in various SGLT2 inhibitor CVOTs.

## Supplementary information

**Additional file 1: Figure S1.** Change of E/e’ for each single patient treated with empagliflozin (n=20; black line) or placebo (n=22; blue dotted line) after 1 day, 3 days, and 3 months.

## Data Availability

All data generated or analysed during this study are included in this published article and its supplementary information files.
