# Supplementary figures and images for: Empagliflozin does not change cardiac index nor systemic vascular resistance but rapidly improves left ventricular filling pressure in patients with type 2 diabetes: a randomized controlled study
Source: Cardiovasc Diabetol. 2021 Jan 7;20:6. doi: 10.1186/s12933-020-01175-5 (PMC7791833; doi:10.1186/s12933-020-01175-5)

## Slide 1
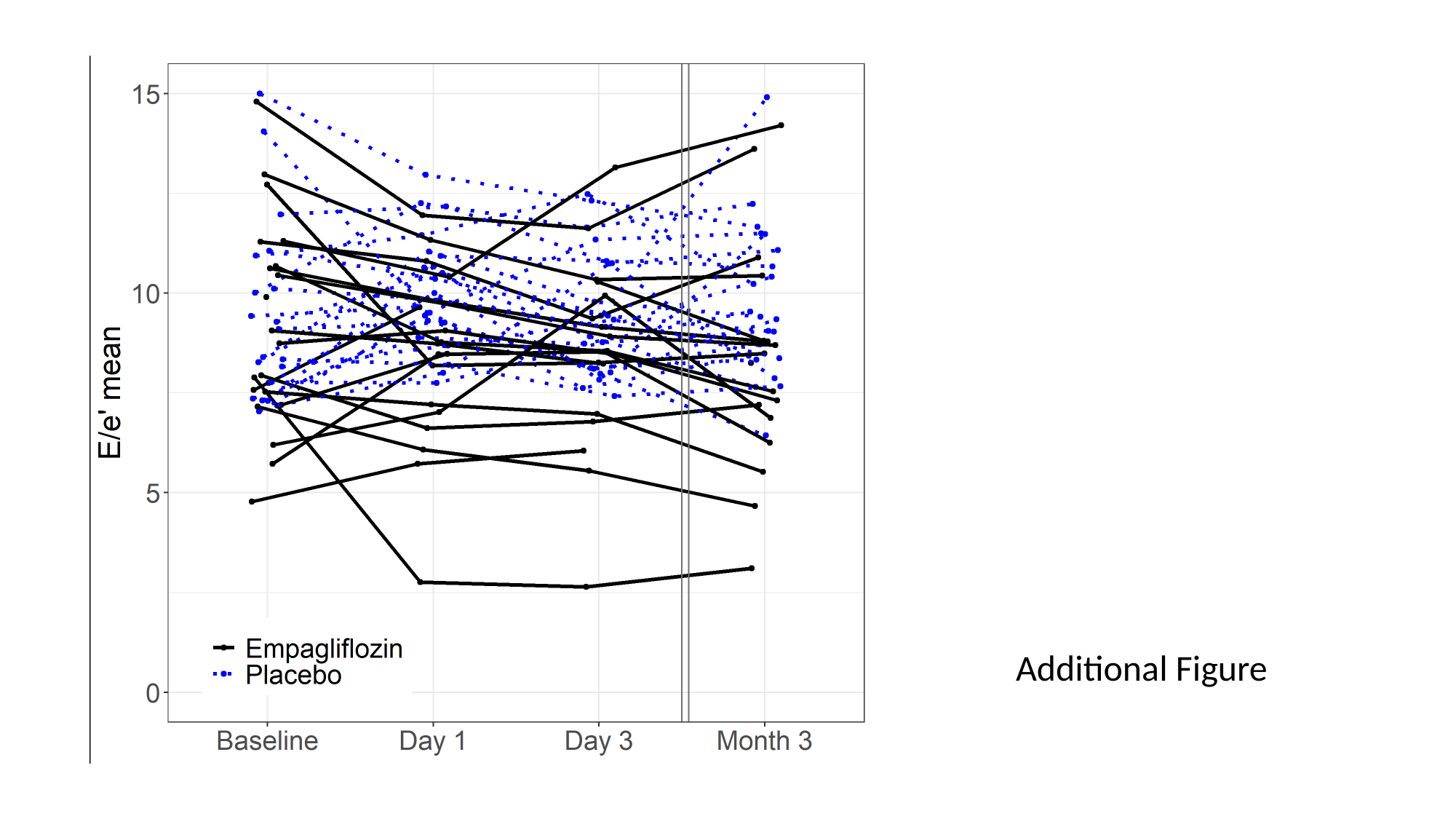

Additional Figure

Supplement: Supplementary file 1 — Additional file 1: Figure S1. Change of E/e’ for each single patient treated with empagliflozin (n=20; black line) or placebo (n=22; blue dotted line) after 1 day, 3 days, and 3 months. [file 12933_2020_1175_MOESM1_ESM.pptx]
